# An Overview on Probiotics as an Alternative Strategy for Prevention and Treatment of Human Diseases

**DOI:** 10.22037/ijpr.2020.112232.13620

**Published:** 2019

**Authors:** Mahdi Taherian, Pariya Mahin Samadi, Hossein Rastegar, Mohammad Ali Faramarzi, Mohammad Rostami-Nejad, Mohammad Hossein Yazdi, Mostafa Rezaei-Tavirani, Zeinab Yazdi

**Affiliations:** a *Food and Drug Laboratory Research Center, Iran Food and Drug Organization (FDO), Ministry of Health and Medical Education (MOH), Tehran, Iran. *; b *Department of Medical Genetics, Shahid Beheshti University of Medical Sciences, Tehran, Iran. *; c *Biotechnology Research Center, Faculty of Pharmacy, Tehran University of Medical Sciences, Tehran, Iran. *; d *Food and Drug Cosmetic Research Center, Iran Food and Drug Organization (FDO), Ministry of Health and Medical Education (MOH), Tehran, Iran. *; e *Department of Pharmaceutical Biotechnology and Biotechnology Research Center, Faculty of Pharmacy, Tehran University of Medical Sciences, Tehran, Iran.*; f *Gastroenterology and Liver Diseases Research Center, Research Institute for Gastroenterology and Liver Diseases, Shahid Beheshti University of Medical Sciences, Tehran, Iran. *; g *Recombinant Vaccine Research Center, Tehran University of Medical Sciences, Tehran, Iran. *; h *Evidence-based Evaluation of Cost-Effectiveness and Clinical Outcomes, The Institute of Pharmaceutical Sciences (TIPS), Tehran University of Medical Sciences, Tehran, Iran. *; i *Proteomics Research Center, Faculty of Paramedical Sciences, Shahid Beheshti University of Medical Sciences, Tehran, Iran. *; j *Department of Medicine and epidemiology, School of veterinary Medicine, University of California, Davis, USA.*

**Keywords:** Probiotics, Cancer, Immune Responses, Treatment, Diabetes mellitus, Multiple sclerosis

## Abstract

Probiotics are viable and useful microorganisms, which are beneficial factors for human and animal health by altering their microbial flora. Most of the probiotics belong to a large group of bacteria in the human gastrointestinal tract. There are several clinical shreds of evidence that show anti-carcinogenic effects of probiotics through altering digestive enzymes, inhibition of carcinogenic agents, and modulating the immune responses in experimental animals. Many studies have been performed to evaluate the potential effectiveness of probiotics in treating or preventing neurological diseases such as MS and novel treatment modality for T1D. The purpose of this study is to have an overview on probiotic microorganisms and to review the previous researches on the effects of probiotics on health through currently available literatures. The study was performed using following keywords; Probiotics, Cancer, Immune system, Multiple Sclerosis (MS) and Diabetes mellitus. PubMed/Medline, Clinicaltrials.gov, Ovid, Google Scholar, and Reaxcys databases used to find the full text of related articles. According to the current available data on probiotics and related health-promoting benefits, it seems that, consumption of probiotics can lead to the prevention and reduction the risk of cancer, diabetes, and multiple sclerosis. Although for the better and more decisive conclusion, there is a need to larger sample size clinical studies with more focus on the safety of these biological agents and their possible beneficial effects on different population.

## Introduction

Live microorganisms, especially the lactic acid-producing bacteria, have been used in foods since 100 years ago for maintaining and improving human health (1, 2). In 76 BC, use of milk fermentation products were recommended by the Romans for treatment of gastroenteritis (1). The first clinical studies on probiotics were performed to evaluate the possible effect of these bacteria on constipation during 1930s. Since then, studies about probiotics in terms of number have steadily increased, in particular in European and Asian countries (3). 

Addition of bacteria to food has a long history and goes back to the beginning of the last century that Metchnikoff conducted important researches in this field at the Pasteur Institute (4). Metchnikoff found that having a long lifespan among the villagers of Bulgaria related to the high consumption of fermented milk (5). 

The aim of the present study is to have an overview on probiotic microorganisms based on currently available literatures related to the effects of probiotics on health condition.

*Probiotic Organisms*

Probiotics are living microbial supplements that exert health benefits through different mechanisms including producing inhibitory compounds and competing with pathogens for binding sites, stimulating and regulating immune response, elevating the capacity of the immune system and improving the microbial balance of the GI tract (6). The definition of probiotics is constantly changing and maturing over time (8). Probiotics were initially introduced as a kind of microbial substances, which could stimulate the growth of microorganisms, while they are currently used for the explanation of prebiotics (8). According to Fuller, probiotics have desirable effects on the host’s body and can survive in the gastrointestinal tract for a long time (9). Potential benefits of probiotic foods on human health make them as a therapeutic option for treating irritable bowel syndrome (IBS), diarrhea, lactose intolerance, and hyperlipidemia. The effects of probiotic foods include improving the balance of gastrointestinal microflora, stimulating the immune system, and lowering serum cholesterol level (10). Previous studies have shown that probiotics also have anti-tumor properties, which are due to the production of secretory enzymes that inhibit the toxicity of carcinogens in the intestine and inhibit the cancer-inducing damages (11). Today, probiotics are not use solely as a growth stimulant but also are used for stimulating the immune system and preventing the spread of many infectious diseases (12).

Until now, various fungal and bacterial microorganisms used for keeping or boosting human health. However, In order to name a microorganism as probiotic, in addition to its possible health-promoting effect, it should be able to survive in the gastrointestinal tract and colonize there. Other important parameters discussed as follow: Among the various microorganisms, which used as probiotics, some were studied and approved including: *Lactobacilli, L. acidophilus, L. bulgaricus, L. casei, L. paracasei, L. heleveticusL. plantarum, L. Sake, Bifidobacterium longum, Enterococcus facium, Lactococcus lactis, E. feacalis, Streptococcus* *thermophiles, Clostridium butiricum, Saccharomyces cerevisiae and S. boulardii *(13). According to some clinical evidences, among the aforementioned probiotic bacteria *lactobacilli* and *Bifidobacterium* have greater effects (14). Moreover, *Saccharomyces boulardi* has the same beneficial effect among yeasts (15). On the other hand, selection of probiotics as a beneficial factor for human health is related to the historical use of them for a long time without any detrimental side effects (16). 

**Figure 1:**
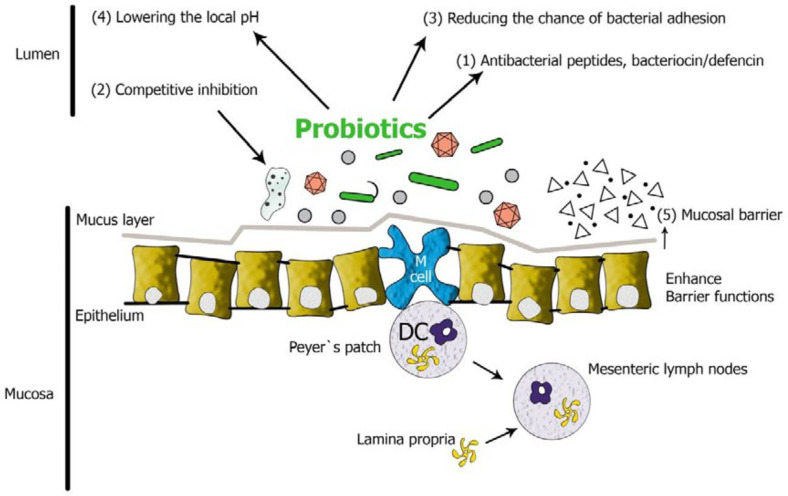
Inhibition of pathogenic bacteria and increased intestinal protection by probiotics; General pattern of interference between probiotics and intestinal mucosa (3)

In general, the required criteria for considering bacterial species as probiotic include: 1) Resistance and survival in preparation stages, 2) Viability and activity in the digestive system, i.e. resistance to gastric acid and bile acids, 3) Capability to adhesion to the intestinal epithelial cells, 4) Ability to cope with pathogens by generating anti-bacterial compounds, competitive elimination or reduction of intracellular pH (Figure 1) (16-18). 

Bacterial probiotics have beneficial effects on their host and can reduce the risk of cancer through reducing the underlying causes of inflammatory diseases and cancers. For instance, they balance intestinal microbial flora, prevent pathogenic bacteria from binding to the intestinal mucosal wall and suppress inflammation. (19,20). Numerous researches were done on probiotics and their effects on human health; however, several types of studies demonstrated these benefits on livestock animals and even aquatic animals. For example; previous studies investigated the beneficial effects of probiotics, especially lactobacilli, on improving growth indices, the performance of the immune system and resistance to diseases in Rainbow trout. In addition to bacterial probiotics, yeasts also cause the same effects in aquatic animals (21, 22). 

Unlike synthetic and chemical drugs that exert their effects through one defined pathway, probiotics have different mechanisms, which multiply their effect and durability in the host. For this reason, probiotics are known as important factors in homeostasis (23). The most important mechanisms involved in the probiotics function and their specific and non-specific benefits listed in Table 1. 

Previous *in-vivo* and *in-vitro* studies have shown that some probiotics have anti-genotoxicity activity. For example; the ability of *Lactobacillus casei* to inhibit DNA damage (Deoxyribonucleic acid) in the nitrous and guanidine mutagens exposed rats’ colon has been already investigated. Previous studies have also shown that *Lactobacillus casei* and *Lactobacillus paracasei* can bind to mutagens of food origin and inactivate them (11-24). Moreover, it was reported that some *Lactobacillus* strains can eliminate the toxicity of carcinogens (25, 26).

*Effects of Probiotics in health maintenance and disease prevention*

Probiotics seem to have promise in the prevention or treatment of several diseases. The following section describes some beneficial effects of probiotic preparations in the prevention, prophylaxis and treatment of human diseases such as colon cancers, diabetes and MS as important human health diseases in the present century.

*Probiotics and Cancer*

**Figure 2:**
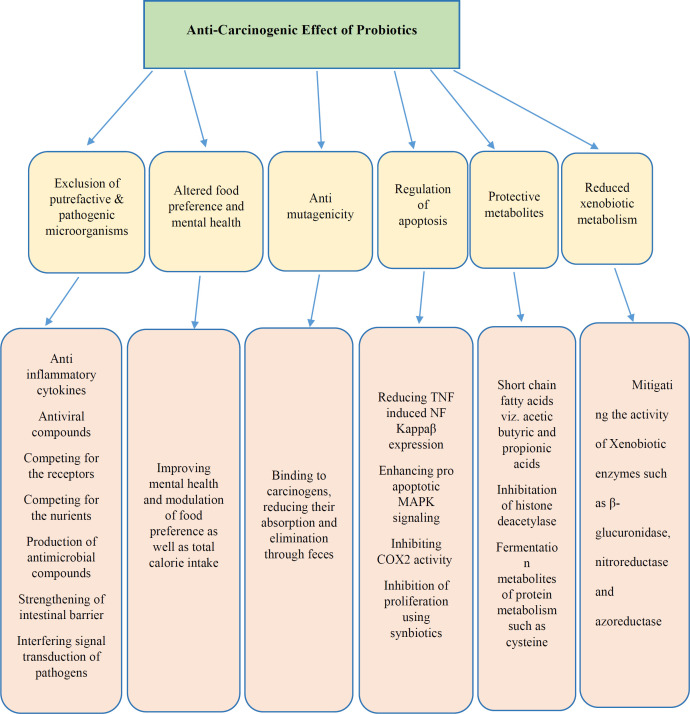
Different effects of probiotics in conferring anticancer property

As per WHO cancer fact sheet, cancer has been a dreadful disease affecting peoples all over the globe. Uncontrolled proliferation and resistance to apoptosis considered as the main features of tumor cells; therefore, an agent that led to apoptosis can be considered as an anticancer factor (27). It is also known that, at least half of all cancers occur due to presence of some compounds in the diets; several molecular and cellular steps in the carcinogenic pathways were defined, and the body of evidence indicates a prominent causative role for environmental factors, including obesity and diet. all of these factors are associated with changes in the gut microbiome. Therefore, Natural agents that have anti-carcinogenic effects, such as probiotics have received prime focus in recent years (28). Accordingly, probiotics are non-pathogenic microorganisms that proved to have anti-cancer activity (Figure 2) (29). 

One of the main known anti-cancer mechanisms of probiotics is done through neutralizing the poisoning materials that led to gene damage in the intestines. The results of an *in-vitro* study using carcinogenic 1 and 2 dimethyl-hydrazine in the mouse colon support this claim (30, 31). Another *in-vitro* studies have demonstrated that probiotic strains, *Lactobacillus fermentum*, *L. acidophilus* and *L. rhamnosus* have potent role in suppressing colorectal cancer cells and promoting normal epithelial colon cell growth through the production of SCFAs (ferulic acid). *L rhamnosus* were found to reduce radiation-induced intestinal damage and apoptosis in the proximal jejunum of mice in a TLR2-, COX2- and MyD88-dependent fashion. Protection can also be mediated through the unusual mechanism of increased migration of mesenchymal stem cells into the lamina propria. 

Recent studies have shown that metabolites, isolated from milk, have a short lifespan and can be fermented by strains like *Lactobacillus bulgaricus* and *Streptococcus thermophiles*, are very effective in terms of inhibiting intestinal carcinogenesis (32). Consumption of probiotics leads to the production of a wide range of fermentation products, such as high concentrations of short chain fatty acids. Totally, these factors reduce the burden of genotoxic factors in the intestine and also, increase the production of factors that can inactivate toxic compounds. For example, Butyrate is one of the protective factors that can reduce the risk of cancer (33, 34). Lactic acid bacteria in fermented milk can have inhibitory effects on the development of carcinogens and tumors in animal models. In addition, *Lactobacillus bulgaricus* and *Lactobacillus plantarum* bacteria which are available in yogurt and are produced as probiotic pills showed good anti-mutagenic effects. These bacteria reduce the absorption of carcinogenic and mutagenic substances through changing the intestinal flora (35). Furthermore, cytotoxic effects of two different probiotic strains *L. acidophilus* and *L. casei* against several colorectal cancer cell lines (e.g. Caco-2 and HRT-18) with *in-vitro* anti-proliferative activity assay. 

Until now, several potential mechanisms of probiotics described including: alterations in microbiota species and metabolism, changes in colonic pH, binding or inactivation of carcinogens, enhancing immune responses, reducing colonic inflammation, decreasing epithelial proliferation, influencing the early phase of tumor growth by reducing the epithelial effect on active carcinogens and increasing apoptosis (36). Previous studies have suggested that the use of probiotics inhibits the primary phase of tumor growth and gut microbiota may affect the immune system systemically (24-37). Yet, the effects of probiotics on other cancer types have also drawn researchers’ attention. In the past decades, some clinical studies were conducted in this area, which are listed in Table 2. 

Like probiotic bacteria, probiotic yeasts have important implications for cancer. In a study, heat-killed *Saccharomyces cerevisiae* caused the cell apoptosis in three different cell lines of breast cancer (MCF-7, ZR-75-1, and HCC70) (38, 39). In other study, *Saccharomyces cerevisiae* injected in mice, with a previously induced tumor by injecting MCF-7 human breast cancer cell line for 45-days, and results showed a significant decrease in tumor volume of treated mice (40). In another study, direct injection of yeast *Saccharomyces cerevisiae* (killed through heating) into the tumor lead to significant retrogression, induction of apoptosis and regulation of the innate immune system (41). It has been demonstrated that the induction of apoptosis by *Saccharomyces cerevisiae* on breast cancer cells depends on intracellular mitochondria calcium release pathway. The decrease in this apoptosis depends on the expression level of Bcl- 2 (Anti-apoptotic gen) and its increase depends on the expression level of Bax (Pro-apoptotic gen) (42).

As previously mentioned, probiotics can have anti-mutagenic effects. For instance, Lactic acid bacteria can prevent colon cancer through mechanisms like changing metabolic activity of the intestinal microflora, changing the physical and chemical conditions of the colon, destroying the carcinogens through attachment and changing intestinal microflora quantitatively and qualitatively. Also, the inhibition of the production of carcinogens such as ammonia and secondary bile acids is another effect of probiotics in gut microenvironment (44). *Bifidobacteria* is one of the most effective probiotics in controlling colon cancer. These bacteria can prevent the growth and proliferation of pathogenic bacteria such as *E.coli* (enterohaemorrhagic *E. coli* (EHEC) strain, extraintestinal E. coli (ExPEC)) and *Clostridium perfringens* in the colon through generating acidity, reducing the intestinal pH and regulating the level of these bacterial enzymes such as beta-glucuronidase, which converts procarcinogens into carcinogens (45). Similarly, the effect of probiotics on reduction of faecal-water-induced DNA damage evidence in colonic epithelial cells was shown. Probiotics were evaluated to help control side effects of radiotherapy and chemotherapy used for the management of intra-abdominal and intrapelvic cancers. These and other studies point to the potential positive effects of probiotics in the improvement of radiation and chemotherapy treatments for cancer patients. One problem with these treatments is damage to the small bowel and large intestine of patients and they have some adverse effects such as incapacitating diarrhoea, dehydration and malnutrition, which can limit the amounts of therapy. But probiotics that effectively mitigate these side effects of cancer treatment could be important therapeutic agents. 

To sum up, considering the available data on probiotics and their anti-cancer properties it can be concluded that these microorganisms can eliminate the risk of cancer by changing the gut microenvironment in terms of colonized or resident microbiome and their metabolites. But, further and more precise studies are still needed and this potential ability of probiotics must be confirmed by *in-vivo* models and proceed towards animal and clinical trials.

*Type 1 diabetes and Probiotics*

Nowadays, it is well demonstrated that Type 1 diabetes (T1D) as an immune related disorder which considered as one of the most common chronic diseases of childhood (52-55). In fact, T1D is a long-term consequence of immune auto-reactivity; which finally leads to almost depletion of pancreatic islets b-cells (56-58). Although, T1D is a multifactorial disorder and genetic predisposition and environmental factors are two important risk factors for people susceptiblity to this disease. These factors trigger the autoimmune responses against B-cells and result in the B-cells destruction, which makes the body dependent on exogenous insulin injection (59-61). 

Comparisons between countries and regions with low and high incidence rates of T1D, convey this fact that, higher levels of urbanization and more restricted sanitary system (which lead to the limited contact with microorganisms) have significant effects on rising the incidence of T1D, however, the results are not clear enough to make a conclusive conclusion (62-64,65). For this reason, whereas the profit of modern life cannot be ignored, it would be better to follow our ancestors’ recipes and food style to enjoy the advantages of traditional life in particular in terms of health. (Probiotics are among the inevitable elements of traditional foods.)

**Figure 3.**
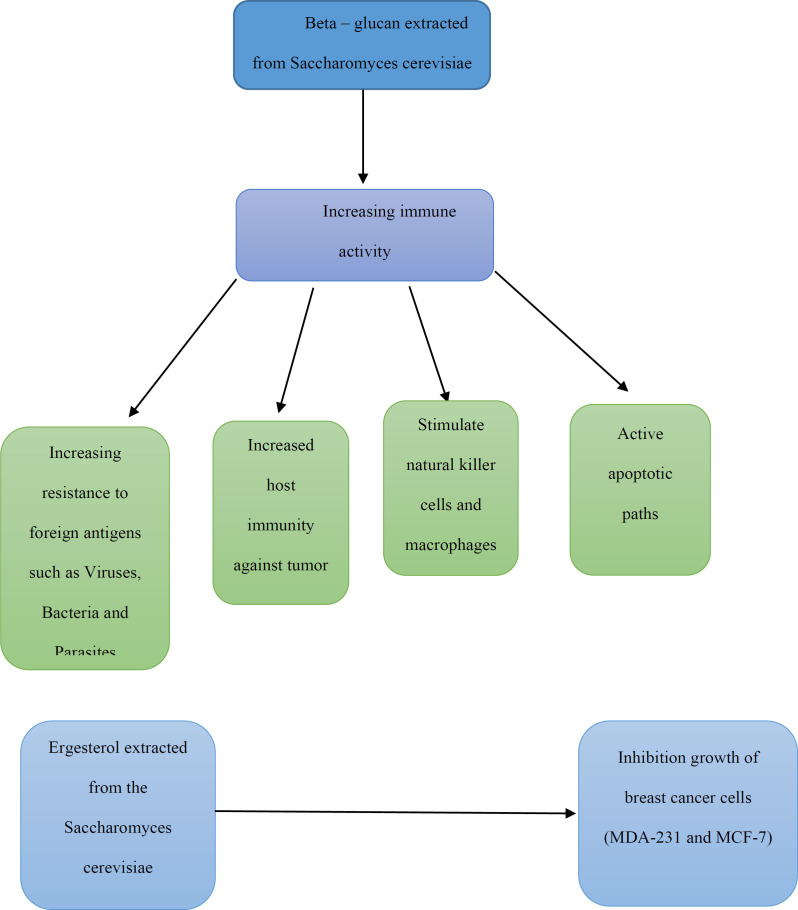
Compounds from yeast and their effects on cancer

In order to confirm probiotics as a possible treatment modality for T1D, many studies used animal models such as diabetes-prone rodents including the NOD (Non-obese diabetic) mice’s and performed many standard clinical studies on infants and children (66, 67). In a study on eight children with T1D compared to controls, it was demonstrated that despite healthy infants, which had healthier and more stable microbiome in their toddler stage, in children who are prone to develop autoimmunity, microbiome is less diverse and stable (68). Another study on 200 subjects represented the fact that using probiotics during the first 6 months of life can decrease the possibility of getting autoimmune diabetes even in infants with genetic predisposition or familial history of this disorder (69). Some of the most important studies collected in Tables 3, 4 and Figure 3. 

As a result of the mucosal barrier disruption, a local pro-inflammatory condition occur which leads to increased antigenic exposure. This aberrant antigenic exposure can be a reason for further autoimmune reaction and development of autoimmune disorder such as T1D (77, 78). This local mucosal pro-inflammatory condition may lead to the distinct attack of pancreatic B-cells, but the mechanism of this process is not completely clear. 

**Figure 4:**
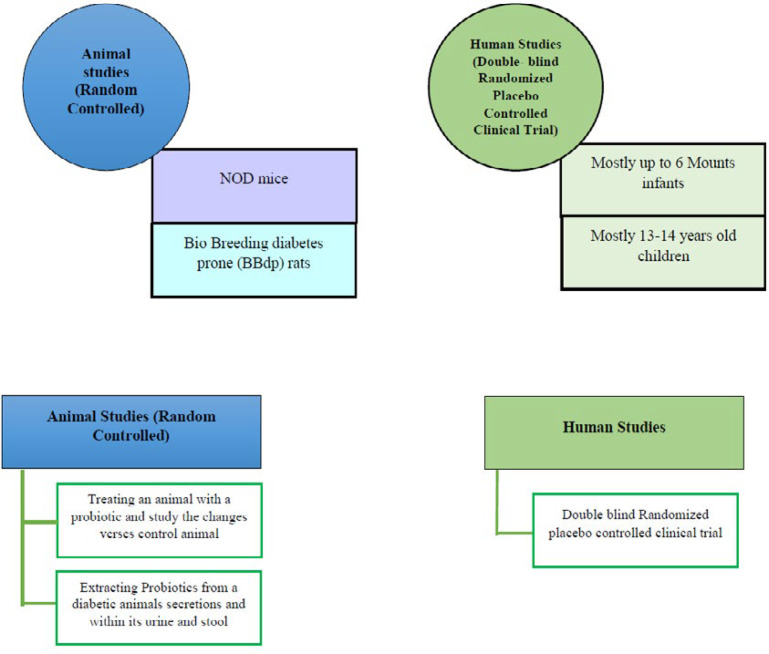
Human and animal studies

**Figure 5:**
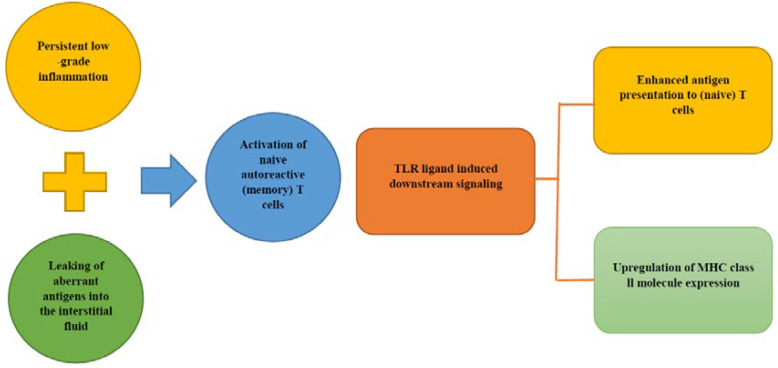
TLR ligand-induced downstream signaling events include upregulation of MHC class II molecule expression and increases the presentation of antigens to (naive) T-cells, therefore initiate the development of antigen-specific immune response

It is thought that the persistent low-grade inflammation in combination with the interstitial fluid exposure to immune-hidden antigens could result in the activation of naive T-cells (79-81). Toll-like receptors (TLRs) are expressed on innate immune cells and play a critical role in the activation of a specific T-cell response (82). Evidence for TLR involvement was provided by the finding that knocking out MyD88 (coding for a protein involved in TLR signaling) in non-obese diabetic /diabetes-prone mice prevented the development of diabetes (83). TLR ligand-induced downstream signaling initiate the development of antigen-specific immune response events include: up-regulation of MHC class II molecule expression and increasing the antigens presentation to (naive) T-cells (Figure 4, 5) (84).

Administration of exogenous insulin is the only proofed available treatment of T1D (85), and efforts to find a lesser interventional cure for T1D have not yet succeeded. Like almost all diseases, T1D prevention is a previous step to treatments (86). 

Recent studies show early dietary intervention and/or direct microbiota manag-ement can also influence the development of T1D (87).

In children with T1D, bacteria that contribute to fermentation of dietary fibers or in the synthesis of short-chain fatty acid SCFA like butyrate, were significantly reduced (88). Butyrate has significant supporting effects on glucose metabolism and on gut barrier healthy function. As recent studies show, this substance can reduce oxidative stress and, also it can mediate inflammatory responses in the gut especially in the intestinal mucosa. This result might shed a light on a way to reach the primary prevention of T1D via gut microbe management and probiotics administration (89, 90).

*Type 2 Diabetes and Probiotics*

Type 2 diabetes (T2D) is known as a metabolic disorder in which blood sugar levels rise due to both insufficient insulin production and resistance to insulin (91, 92). T2D has a higher prevalence compared to T1D and accounts for 85% to 95% of all cases of diabetes. The prevalence of this disorder is almost 8% among the Iranian adult population (93). As mentioned earlier, since decades ago, the incidence of diabetes and obesity dramatically increased, as far as it can be considered as a worldwide epidemic (94). 

Changing in dietary habits, in particular increased consumption of lipids, which can cause low-grade inflammation, proposed to be responsible for the dramatic rise in metabolic disorders. This issue has particular importance in life threatening metabolic disorders like cardiovascular disease and/or coronary artery disease (CAD) (95, 96). 

Abnormal metabolic profile, is not the only complication of T2D, rather, chronic inflammations, increased level of pro- inflammatory cytokines as well as oxidative stress have critical roles in T2D process and disease prognosis (97, 98). Accordingly, however B-cell dysfunction and/or insulin resistance are important events in T2D, but without paying attention to the role of the inflammatory status in this disorder, treatments will not result in proper outcomes (99). It is observed that consumption of herbal medicines, omega-3 fatty acid supplements, antioxidants, E, A, and C vitamins besides coenzyme Q 10, can help reduce oxidative stress. Furthermore, non-steroid anti-inflammatory medicines like aspirin (in low doses) and statins are recommended for controlling the patients’ condition. 

Moreover, probiotics can also play a useful role in this complicated disorder, not only for their ability to reduce inflammation and oxidative stress markers, but also for their impact on the improvement of glycemic and insulin metabolism (100-104). 

Alterations in the gut microbiome affect both intestinal peristalsis and the expression of several genes involved in gut microenvironment health and function (105). It also has an impression on the development of the enteric nervous system, development and function of mucosal immunity and enzymatic de-conjugation of bile acids. Additionally, alterations in the gut microbiota play a role in reducing oxidative stress and inflammation through the conversion of cholesterol into coprostanol and increasing glutathione (GSH) levels, scavenging free radicals and reducing the interleukin-6 (IL-6) level in adipocytes (106). 

Results of some studies in T2D patients compared with healthy people shows that, diabetic patients have a significantly lower number of butyrate producing bacteria (90). Regarding to the results of these studies, it is proposed that the consumption of probiotics would help reduce pro-inflammatory factors (108, 109). By collecting the results of recent studies, some of the possible mechanisms linking probiotics to diabetes can be proposed as follow:

Butyrate is a short-chain fatty acid (SCFA) that has a major role in providing energy for intestinal cells (90). There is also a possibility that SCFAs can directly prevent the early stages of inflammation, which is connected with T2D. Studies demonstrated that low-grade systemic inflammation can be induced by the fragments from Gram-negative bacteria living in the gut (called endotoxins), which can cross the intestinal mucosa and enter the blood circulation. Probiotics can prevent the translocation of pro-inflammatory lipopolysaccharides into the bloodstream, thus can inhibit related inflammatory responses. Results of some recent clinical studies have shown the higher level of butyrate-producing bacteria after infusion of feces from lean donors in insulin-resistant men with metabolic syndrome, which was accompanied by beneficial metabolic effects (66,110-113). 

The role of the intestinal microbiota in obesity and weight gain and development of adipose tissue was observed in a recent study in which germ-free mice were colonized with the gut microbiota from genetically obese ob/ob mice and gained more weight (114, 115). 

TNF-α which is released constantly from adipose tissue, is another important mediator for inflammatory response in those individuals without healthy bacteria in gut microenvironment (112). 

It is also demonstrated that during high fat diet-induced diabetes, normal intestinal flora translocate in a pathological manner from the intestine towards the tissues and trigger a local inflammation by which diabetes can be emerged? (116, 117). 

Altogether, considering aforementioned hypothetical and demonstrated mechanisms, probiotic can be a good option for T2D patient’s health care. 

The study designed by Honda and colleagues, showed the lactic acid bacteria can confer an anti-diabetic effect in both normal and type 2 diabetic mice (118). Meanwhile, in the clinical study conducted by Ejtahed and colleagues on 60 men and woman, it was shown that, probiotic yogurt can have antioxidant effect in type 2 diabetic patients and have some beneficial therapeutic outcomes (119). 

*Probiotics and Multiple sclerosis*

Multiple sclerosis (MS) is an autoimmune, inflammatory and demyelinating disease, which is associated with the destruction of nerve cells insulating covers in the brain and spinal cord and eventually leads to axonal degeneration and neuronal death. This disease is one of the main reasons of neurological disabilities in young adults that its definitive cure is not yet identified and the common therapeutic approach for these patients are expensive therapies with low efficiency (120). Therefore, researchers, clinicians, and pharmaceutical companies are currently investigating new therapeutic methods and medicines for MS treatment (121). Currently, a substantial interest is dedicated to discovering the potential therapeutic and preventing effectiveness of probiotics in a wide range of neurological diseases such as MS (122). An increasing number of evidence confirmed that the gut microbiota influences the human brain development and its activities (123). One of the earliest preclinical studies to evaluate probiotic efficacy in the preventing and treatment of neurological diseases was performed by Goudarzvand and his team. The results of this study showed that the anti-apoptotic role of E and D3 vitamins in the hippocampus of the demyelinated rat, induced by the injection of ethidium bromide (EB) (124). 

Evidence suggests that many MS patients develop iron deficiency anemia (125). Due to the importance of iron in the biosynthesis of cholesterol and lipids, which are the main constituents of myelin, iron deficiency can attenuate the myelination process by reducing the oligodendrocyte-myelin cells (126). Various studies considered the mechanism of probiotics to increase iron absorption and as results to a reduction in the MS complications. Recently, the role of vitamin D3 identified in the improvement of demyelinated rats (124, 127). Hill *et al*. observed an increase in vitamin D in both sexes with consumption of the *Lactobacillus salivarius* (128). Based on the collected data, using probiotics can be effective in increasing the effect of recovery factors affecting remyelination process including vitamin D3. 

It has been reported that *Lactobacillus plantarum* and *Lactobacillus paracasei* can suppress the succession of mice-developed experimental autoimmune encephalomyelitis (EAE), downregulate myelin oligodendrocyte glycoprotein MOG)-reactive Tcells and also can change the central immune response from Th1 to Th2 (129). However, a more comprehensive understanding about their method of action is essential to attribute the role in improving neurological manifestations or declining the occurrence of neurodevelopmental disorders to them (130). The intestinal microbiota has an intense influence on several neuromodulators and neurotransmitters such as GABA, serotonin, monoamines, and brain-derived neurotropic factors, which transfer signals to the brain through different ways, such as the entero-chromaffin cells, the enteric nerves, and the systemic circulation across the blood-brain barriers. In these conditions, permeability appears to be controlled by the microbiota in experimental models (131).

*Pharmacokinetic and Safety of Probiotics*

Similar to any other biopharmaceutical, probiotics have their own characteristics as a medicine. Due to their direct contact to gastrointestinal tract, and their acid resistance, probiotics are considered as a good delivery system to this organ (132). So far, most of the pharmacological studies on probiotics and their pharmacokinetic profile investigated the stability of these bacteria in gastrointestinal environment and assessed their survival (133). As mentioned before, probiotics need to be attached to the epithelial cells, colonized and survived in GI tract to confer the health beneficial effects. These features can be determined in both *in-vitro* and *in-vivo* studies and can be used as good parameters for evaluation of the potency of this biopharmaceutical. The concentration of probiotics is also a matter of concern for getting the health beneficial effects. Although this is, vary from strain to strain, but the best concentration of probiotics in GI tract is approximately ≥10^6^ colony-forming units/mL (cfu) in the small bowel and ≥10^8^ cfu/g in the colon (134). 

Besides prophylactic and therapeutic effects of probiotics in various diseases, some animal and clinical trials showed that, similar to any other medicine, the adverse effect concern is also associated with probiotic administration. Although, systemic infections, detrimental metabolic products or activities, extra immune stimulation and risk of autoimmunity in susceptible individuals and possibility of gene transfer are not usually recorded as side effects of probiotics according to FAO/WHO (2002). Ideally, in a clinical practice the side effects of probiotics should not recorded more than what observed in the placebo group. While, sever side effect of probiotic in particular in normal individuals are not usual, moderate level of side effects of probiotics in the gut microenvironment are most common. The symptoms, such as constipation, abdominal discomfort, bloating, flatulence, dyspepsia, nausea, are among these moderate side effects. However, for safety concerns like genetically modified organisms (GMO), probiotics foods and product should have a label. 

Imprecise and, even worse, overestimation and propaganda about probiotic may increase the risk of unwanted and harmful side effects especially in abnormal individuals with some chronic and /or underlying diseases (135). 

Results of some clinical and animal trials raise the safety concern about probiotics by showing the high probability of mortality rate in severe acute pancreatitis patients and, the risk of virulence factors transfer to pathogenic or opportunistic pathogens. 

One proposed reason for the convenient prescription of probiotics is related to this fact that, many healthcare practitioners and even consumers have a positive attitude towards probiotic products and do not consider them as a “drug”. Whereas, like any other pharmaceutical product, the safety and efficacy of probiotics should be determined through considering the amount and dosage of the probiotic, the characteristics of the consumer, and the reason for taking probiotic (136). Although, serious hazards of probiotics are unusual, but there are some, and probiotic related septicemia and endocarditis were reported in rare cases of the infection (137). However, results of several studies have shown the safety profile of probiotics in normal individuals. For instance, microencapsulated *L. reuteri* NCIMB 30242 in a yogurt formulation was prescribed to 120 subjects for 6 weeks to treat hypercholesterolemia and results showed that twice-daily dose (5×10^10^ cfu) of this probiotic was safe and can be tolerated. Also, supplemented yogurt with probiotics in patients with IBD was consumed for 6 months and there were no any observed adverse effects during consumption. 

Safety evaluation studies were done in clinical practice and the results were used for the safety analysis of probiotics. Of the eleven studies, seven did not report the adverse events (AEs), whereas four studies reported different grade of unwanted side effects. 

Moreover, regarding to the importance of our gut normal flora as a forgotten organ, one of the big concerns on safety of probiotics is related to their virulence factors, which due to the subject association in gene transfers may result in antimicrobial resistance in intestinal bacterial populations. 

Taken together, due to the lack of adequate clinical trials on the safety of probiotics, there is still no any powerful justification in this regard. Therefore, clinical specialists are highly recommended to carefully consider patients’ conditions before any recommendation of this healthy bacteria, especially in high-risk subjects like newborns and elderly people.

**Table 1:** Benefaction and mechanism of Probiotics for preventing diseases and human health

**Benefits**	**Mechanism of effect**
Production of hydrogen peroxide Production of bacteriocin Production and release of non-bacteriocinic organic metabolites Result: Destruction, prevention of growth and proliferation of other microorganisms in the ecological environment of probiotics	Production of antimicrobial agents
Probiotics which can bind to host cells, prevent pathogens binding to cellular receptors and infection.	Competition over binding to host cell receptor
Consuming essential nutrients such as vitamins needed for pathogens to grow and proliferate	Competition with pathogens on existing nutrients
The receptor degradation meaning failure of pathogenic bacterial or toxic metabolites to adhere to target cells	Ability to alter the specific receptors of pathogens existing on the surface of the host cells
Production of acids to acidify the environment to prevent growth of pathogens sensitive to the acidic environment	Decreasing Environment pH
Regulating the production and secretion of cytokines, induction of non-specific immune responses (Complement system, phagocytosis), induction of specific immune responses (humoral and cellular immunity)	Increasing specific and non-specific immune responses
Converging large molecules into their components and facilitate digestion and absorption of them by the host	Helping in food absorbance
Important role in host biochemical activity	Production of vitamins
Convert lactose to Glucose/Galactose	Lactose tolerance
Enhancing immunity and inhibiting enzymes involved in tumorization	Anticancer
Lowering blood cholesterol and harmful lipoproteins levels. Controlling the transfer of bacterial endotoxins from the intestines to the bloodstream	Reducing blood cholesterol
Reduction of lipopolysaccharides and pro-inflammatory cytokines in the bloodstream. Reducing inflammation; hence, reducing insulin resistance and preventing the destruction of pancreatic beta-cells	Controlling type 2 diabetes

**Table2:** Some clinical studies about effects of Probiotics on cancer

**Results**	**Probiotic/ prebiotic**	**Subject of the study**	**Groups**
Decrease in urine carcinogenicity	*L. casei*	Carcinogenicity of urine after consumption of fried cow meat	6 healthy people
Excretion of urinary mutagenesis factors is 50-70%	*L. acidophilus*	Carcinogenicity of urine and stool after consumption of fried cow meat	11 healthy people
The presence of lactobacilli that reduces inflammation of the rectum	*L. acidophilus* and *B. bifidum*	Cell proliferation in rectal mucosal biopsy	20 patients with adenoma of the large intestine

**Table 3.** Studies conducted on diabetes induced animals

**Results**	**Probiotic/ prebiotic/Bacteria**	**Subject of the study**	**Groups**
*M. avium *infection induced a sustained enhancement in splenic leukocytes , T cells and B cells of NOD mice.	*M. avium*	Changes in B and T Lymphocytes Associated with Mycobacteria-induced Protection of NOD Mice from Diabetes(4)	Female NOD and NON mice (*n*=6) were infected at 2 months of age,
study shows that a streptococcal preparation (OK-432) prevents diabetes in BB rats by suppressing insulitis, which is a morphological expression of anti-islet autoimmune reactions.	streptococcal preparation (OK-432)	Treatment with streptococcal preparation (OK-432) suppresses anti-islet autoimmunity and prevents diabetes in BB rats.(5)	Age-matched BB rats, consisting of 11 males and 14 females
Germ-free NOD female mice have increased diabetes incidence	Restricted Flora Not Germ-Free Condition	The Incidence of Type-1 Diabetes in NOD Mice Is Modulated by Restricted Flora Not Germ-Free Conditions(6)	10 females and 5 male NOD mices
Diabetes prevention after intraperitoneal administration of OM-85.	OM-85 extracts of eight kinds of bacteria	Transforming Growth Factor- and Natural Killer T-Cells Are Involved in the Protective Effect of a Bacterial Extract on Type 1 Diabetes(7)	3 experiments with 6 , 5, and 10 NOD mice’s
the composition of the gut flora is indeed involved in the development of type 1 diabetes	Fecal flora	Antibiotic treatment partially protects against type 1 diabetes in the Bio-Breeding diabetes-prone rat. Is the gut flora involved in the development of type 1 diabetes?(8)	3 experiments based on BB-DP rats spontaneously develop diabetes from 65 days of age on a conventional plant-based (CON) diet.
higher abundance of *Lactobacillus*, *Bacteroides* and *Bifidobacterium* in NOD rats	*Lactobacillus*, *Bacteroides* and *Bifidobacterium*	Culture-independent identification of gut bacteria correlated with the onset of diabetes in a rat model(9)	2 experiments 3 and 10 rats
Treatment which was administered to diabetic rats reduced the elevated blood glucose levels by up to 2-fold.	*Lactobacillus acidophilus,* *Bifidobacterium lactis, and Lactobacillus rhamnosus*	Probiotic treatment reduces blood glucose levels and increases systemic absorption of gliclazide in diabetic rats(10)	10 diabetic rats

**Table 4:** Studies conducted on newborns with a risk of T1D

**Probiotic/ prebiotic/Bacteria**	**Subject of the study**	**method**	**Groups**
*Lactobacillus rhamnosus* GG (5 × 10^9^ cfu), Lactobacillus rhamnosus LC705 (5 × 10^9^ cfu), Bifidobacterium breve Bbi99 (2×10^8^ cfu), and Propionibacterium freudenreichii ss	Probiotics for the Prevention of Beta Cell Autoimmunity in Children at Genetic Risk of Type 1 Diabetes—the PRODIA Study(11)	double-blind randomized placebo controlled study.	264 children
Lactobacillus rhamnosus GG (5 × 10^9^ cfu), Lactobacillus rhamnosus LC705 (5 × 10^9^ cfu), Bifidobacterium breve Bbi99 (2×10^8^ cfu), and Propionibacterium freudenreichii ssp. Shermani JS (2×10^9^ cfu).	Probiotics for the Prevention of Beta Cell Autoimmunity in Children at Genetic Risk of Type 1 Diabetes—the PRODIA Study(11)	double-blind randomized placebo controlled study	200 children with risk of T1D

## Conclusion

Probiotics represent a new area of research in medicine, the examination of the close relationships between food and health. These have attracted intense interest from clinical nutritionists, scientists, and industrialists to work in a collaborative manner to bring down the disease and develop an effective drug with minimal or no side effects. 

Regarding to the available data on probiotics, their direct effects on GI tract mechanisms, and also clinical and animal reports on their related health-promoting benefits, it can be deduced that, this type of medication can be helpful in reducing the risk of diabetes and moderating its related complications. Probiotics can also prevent some kind of cancers specially colon cancer through different ways, including: enhancing the mucus barrier, up regulation of immunoglobulins such as IgA, down regulation of inflammatory cytokines, creating an unfavorable environment through secretion of antimicrobial factors such as bacteriocin, NO, defensing and H2O2. Moreover, probiotics may be beneficial in reducing the inflammation in multiple sclerosis patients.

For the better justification and more decisive conclusion, numerous randomized clinical studies with larger sample size and more focus on the safety of these biological agents are still needed to help determine the potential of probiotics in preventing and treating various diseases. 

Meanwhile, regarding to the strain specific effect of these bacteria and also their diversity in different population with variety of genetic and environmental factors, another important remained concern is that whether prescription of an effective strain of probiotic in a defined medication or not. It may be reasonable that take an extra conservatism about probiotics and their benefits and this medication should not be prescribed worldwide equally.

This review and preliminary data obtained from various research laboratories, provides a basis for suspecting that interventions targeting the microbiota may be effective. However, different randomized clinical studies will be required to clearly establish the potential of probiotics in preventing and treating various diseases.
